# Unmasking photogranulation in decreasing glacial albedo and net autotrophic wastewater treatment

**DOI:** 10.1111/1462-2920.15780

**Published:** 2021-09-28

**Authors:** Chul Park, Nozomu Takeuchi

**Affiliations:** ^1^ Department of Civil and Environmental Engineering University of Massachusetts Amherst Amherst Massachusetts 01003 USA; ^2^ Department of Earth Sciences, Graduate School of Science Chiba University Chiba 263‐8522 Japan

## Abstract

In both natural and built environments, microbes on occasions manifest in spherical aggregates instead of substratum‐affixed biofilms. These microbial aggregates are conventionally referred to as granules. Cryoconites are mineral rich granules that appear on glacier surfaces and are linked with expanding surface darkening, thus decreasing albedo, and enhanced melt. The oxygenic photogranules (OPGs) are organic rich granules that grow in wastewater, which enables wastewater treatment with photosynthetically produced oxygen and which presents potential for net autotrophic wastewater treatment in a compact system. Despite obvious differences inherent in the two, cryoconite and OPG pose striking resemblance. In both, the order Oscillatoriales in Cyanobacteria envelope inner materials and develop dense spheroidal aggregates. We explore the mechanism of photogranulation on account of high similarity between cryoconites and OPGs. We contend that there is no universal external cause for photogranulation. However, cryoconites and OPGs, as well as their intravariations, which are all under different stress fields, are the outcome of universal physiological processes of the Oscillatoriales interfacing with goldilocks interactions of stresses. Finding the rules of photogranulation may enhance engineering of glacier and wastewater systems to manipulate their ecosystem impacts.

## Introduction

**Fig. 1:**
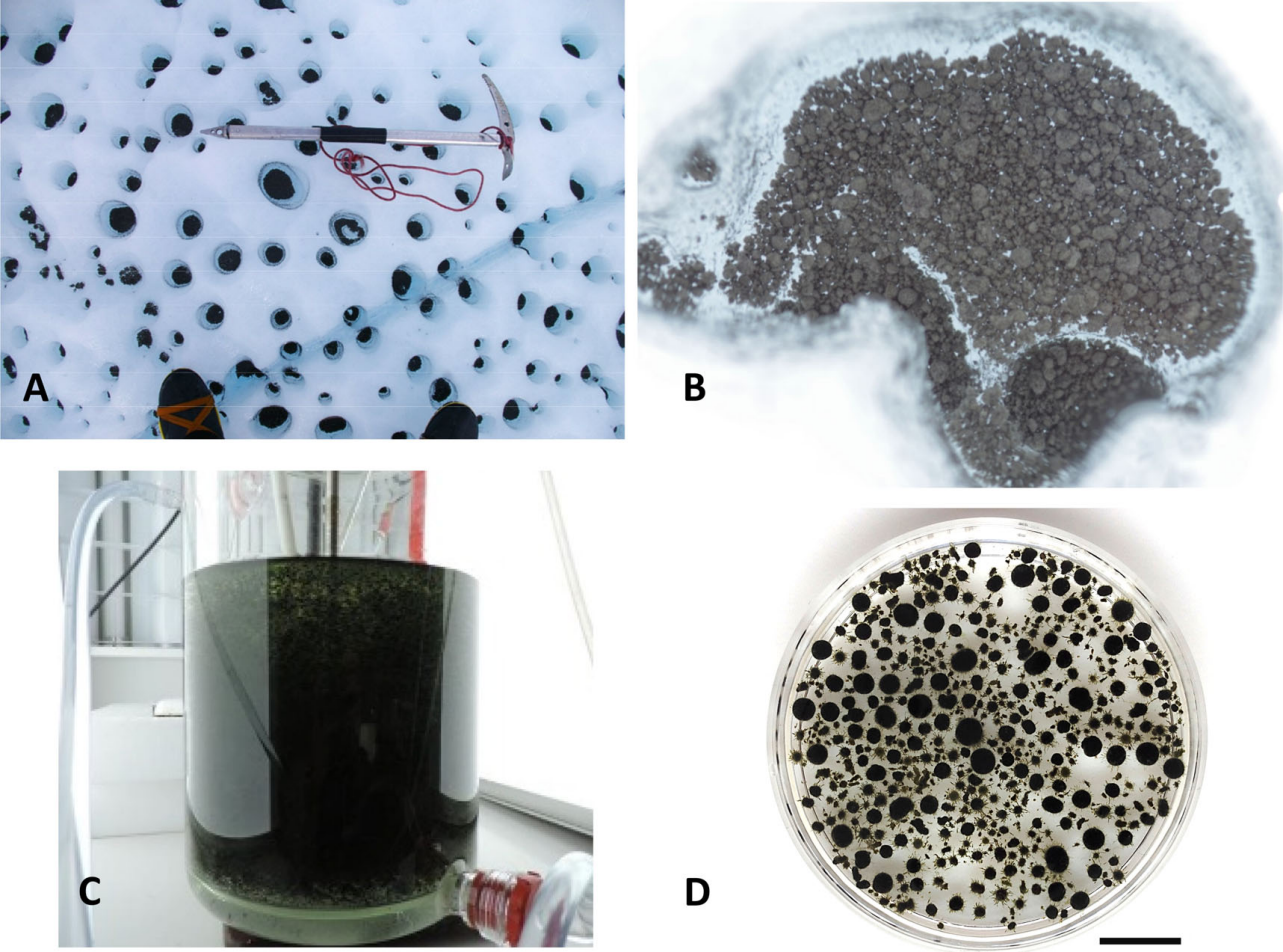
Cryoconites and oxygenic photogranules (OPGs). A. Melt holes, also known as cryoconite holes, on a glacier surface on the Greenland Ice Sheet (August 2012). B. Cryoconites at the bottom of a single melt hole. C. An OPG reactor treating municipal wastewater without mechanical aeration. D. The OPG reactor's mixed biomass. Scale bar: 1 cm.

**Fig. 2:**
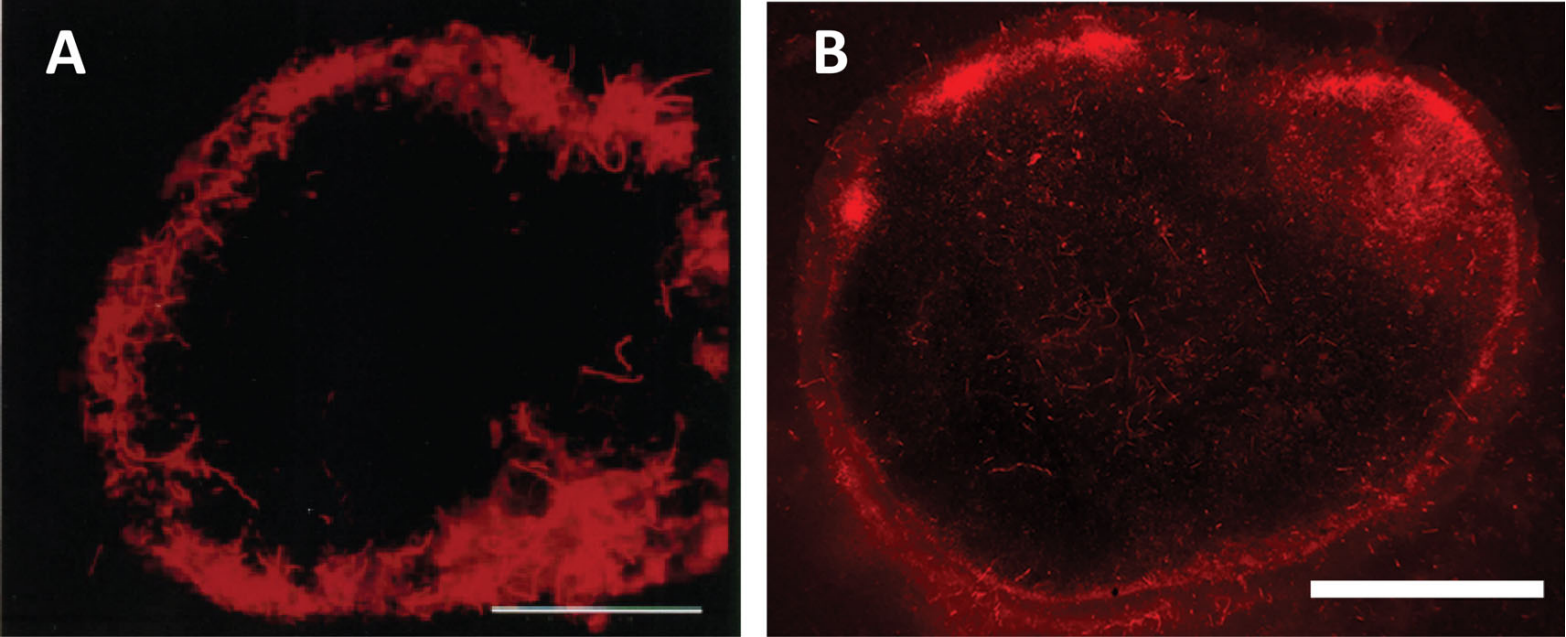
Resemblance in structural formation of cryoconite and OPG revealed by autofluorescence microscopy of cyanobacteria. A. Cross‐section of an Asian alpine cryoconite from Yala Glacier in Nepal (Takeuchi *et al*., 2001). B. Cross‐section of a hydrostatically formed OPG generated from a French activated sludge inoculum (Milferstedt *et al*., 2017). Scale bars for panels are A: 0.5 mm; B: 5.5 mm.

Cryoconites and oxygenic photogranules (OPGs) are quasi‐spherical microbial aggregates that inhabit two extremely different environments on Earth (Fig. 1). The former occur on surfaces of Polar and alpine glaciers and ice sheets and are rich in minerals (>85% by wt.) (Takeuchi *et al*., 2001; Cook *et al*., 2016a; Baccolo *et al*., 2020). The latter, conversely enriched with organic matter (>85% by wt.), grow in wastewater treatment systems (Milferstedt *et al*., 2017; Abouhend *et al*., 2018). Although the difference inherent in the two is therefore obvious, recent studies have recognized their remarkable similarities with respect to granular morphology, structural formation and key microbial group (Milferstedt *et al*., 2017; Stauch‐White *et al*., 2017; Kuo‐Dahab *et al*., 2018; Abouhend *et al*., 2020). In both, mat‐forming filamentous cyanobacteria envelop inner materials forming a spheroidal aggregate (Fig. 2). This network of filamentous organisms and extracellular polymeric substances (EPS) stabilizes a granular habitat by interconnecting with other microorganisms and mineral particles (Takeuchi *et al*., 2001; Langford *et al*., 2010; Cook *et al*., 2016a; Milferstedt *et al*., 2017; Kuo‐Dahab *et al*., 2018).

Since the first report in the 19th Century during exploration in Greenland (Nordenskiöld, 1875), granular and biogenic characteristics of cryoconites have been documented with their presence believed to promote glacial melt. However, only recently we have learned that granular structure of these glacial aggregates is formed by filamentous cyanobacteria (Fig. 2A) and the granules' EPS, especially humified organic matter, absorb significantly more sunlight than surrounding ice (Takeuchi *et al*., 2001). The spectral albedo of ice surfaces covered with cryoconites was 5%–15%, significantly lower than 40%–55% from clean bare ice surfaces (Kohshima *et al*., 1993; Takeuchi *et al*., 2001). Absorbing light and associated heat, cryoconites induce the formation of melt holes (Wharton Jr. *et al*., 1985) – the formed holes themselves extend surface albedo reduction – and grow further beneath quiescent water of the holes (Fig. 1A and B) (Yoshimura *et al*., 1997; Takeuchi *et al*., 2001). Cryoconites get redistributed over the ice surface as the holes collapse during the melting season (Hodson *et al*., 2008; Takeuchi *et al*., 2018) while their dense granular feature helps them to avoid washout in ablation (Takeuchi *et al*., 2001). Cryoconites and cryoconite holes are estimated to cause up to 20% of Polar glacial runoff (Fountain *et al*., 2004, 2008; Tranter *et al*., 2005; Musilova *et al*., 2016). Climate change, especially Polar amplification (Stuecker *et al*., 2018), is expected to expand cryoconite‐induced surface darkening, enhancing melt in the future (Musilova *et al*., 2016; Takeuchi *et al*., 2018).

**Fig. 3:**
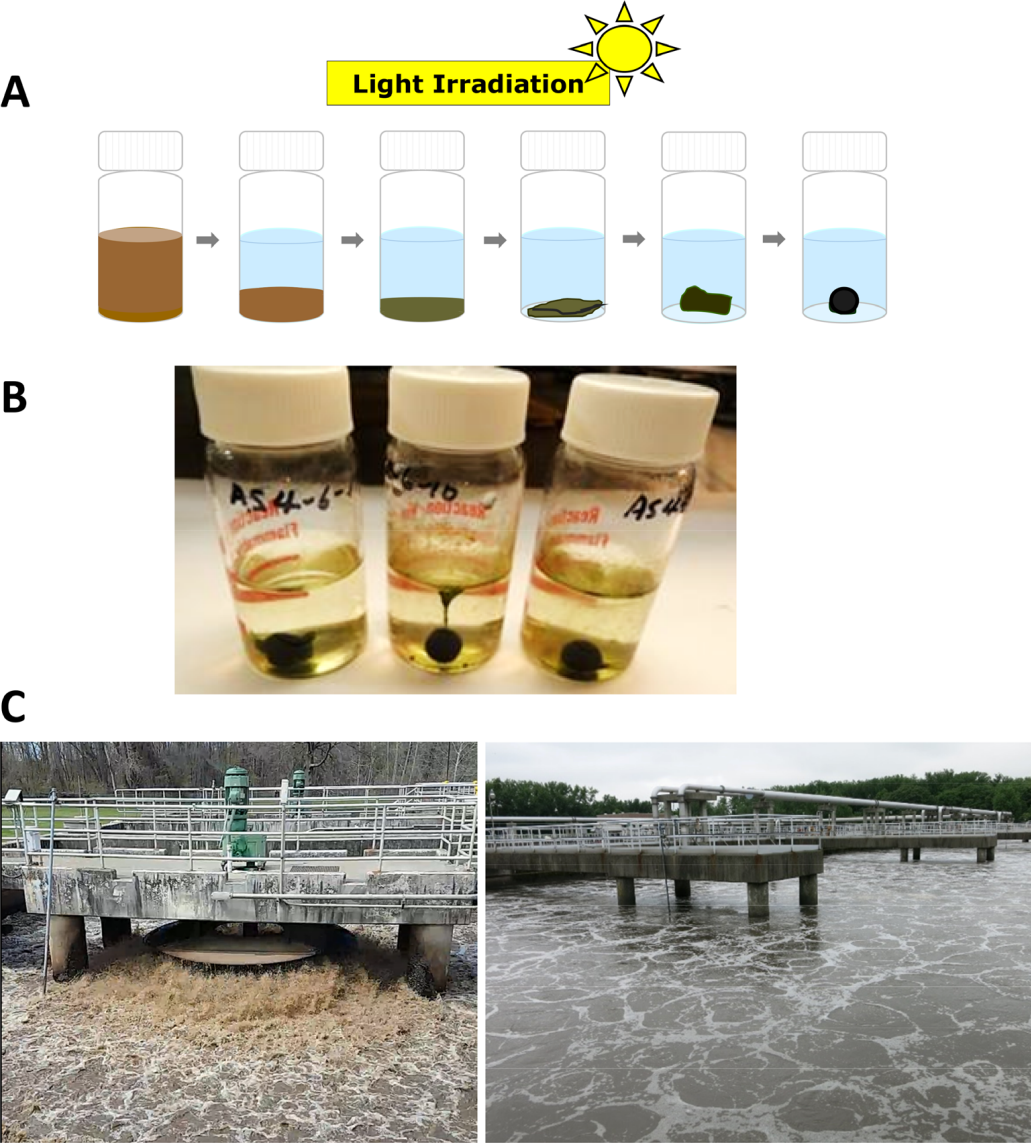
Hydrostatic granulation of OPG from activated sludge inoculum. A mixed liquor of activated sludge obtained from the aeration basin of wastewater treatment plant is inoculated in a closed 20 ml glass vial and placed under illumination with no agitation at room temperatures. This cultivation typically generates a single OPG in several days to a few weeks. A. Schematic of progression in the formation of an OPG in hydrostatic cultivation. B. Hydrostatic OPGs formed from the same activated sludge inoculum. C. Aeration basins of the activated sludge process; aeration and mixing are provided by a mechanical mixing system (left) or a submersed air diffusing system (right).

The reports on OPGs followed a serendipitous discovery in 2011 that activated sludge in a closed 20 ml vial left on a lab windowsill changed to a granule (Fig. 3) (Milferstedt *et al*., 2017; Park and Dolan, 2019). This ‘hydrostatic photogranulation’ occurred with the enrichment of Oscillatoriales, often found in low abundances or undetected in activated sludge (Milferstedt *et al*., 2017; Stauch‐White *et al*., 2017). Reactors seeded with hydrostatically formed OPGs and operated with mixing and illumination were used to treat wastewater without aeration – the aeration alone accounts for 50%–60% of energy for wastewater treatment (U.S. Department of Energy, 2017) or >15% of energy used by municipalities [wastewater treatment plants in the U.S. account for a third or more of municipal energy consumption (U.S. Department of Energy, 2021)]. In reactors new OPG biomass rapidly propagated, concomitantly occurring with chemical oxygen demand (COD) removal and nitrification (Abouhend *et al*., 2018). Proximal growth of phototrophic and heterotrophic microbes within a granule favours carbon fixation during wastewater treatment, leading to net autotrophy (Milferstedt *et al*., 2017; Abouhend *et al*., 2018). Granular biomass enables effective solid/liquid separation, a conventional challenge in both activated sludge and algae‐based wastewater treatment, hence showing potential to make wastewater systems compact.

*How can granules with considerable similarities be formed in these barely related environments?* Notwithstanding their differences, similarities between cryoconite and OPG suggest that a core granulation mechanism is conserved between them. In this interdisciplinary review work, we explore this granulation mechanism by conducting intra‐ and intercomparisons of the two granule systems. Selection of Oscillatoriales, and co‐occurring microbial communities, in cryoconites and OPGs, was studied. We also researched on stresses prevalent in each granule niche and interrogated how they can be related in forming similar granular products conquering vast differences in macroenvironments.

## Selection of Oscillatoriales in cryoconites and OPGs

A strong converging point for cryoconites and OPGs is the ubiquity and abundance of Oscillatoriales. These cyanobacteria have been found as the most abundant group of the Cyanobacteria phylum, not infrequently among entire bacterial phyla (Milferstedt *et al*., 2017; Stauch‐White *et al*., 2017; Uetake *et al*., 2019). Oscillatoriales make up the subsection III genre of Cyanobacteria based on Bergey's bacterial taxonomy (Castenholz *et al*., 2015). Oscillatoriales are unbranched filamentous but distinguished from other groups of filamentous cyanobacteria, i.e. the subsections IV and V cyanobacteria, by being non‐heterocystous and mostly motile (Stal, 2007; Castenholz *et al*., 2015). The subsection IV cyanobacteria, Nostocales, have been found in some cryoconites and OPGs, but their presence was sporadic and mostly with less population than Oscillatoriales (Milferstedt *et al*., 2017; Segawa *et al*., 2017; Uetake *et al*., 2019).

Oscillatoriales are not, however, selected at genus or family level across these granules, although cryoconites' geographical and in OPGs both source‐ and variable‐anchored dominance is present (Table 1). An investigation of 15 Polar and Asian alpine glaciers revealed biogeographical patterns of Oscillatoriales in cryoconites (Segawa *et al*., 2017). The Oscillatoriales in this study were grouped into 15 operational taxonomic units (OTU), which accounted for 87% of total number of cyanobacterial OTUs. In cryoconites from the Arctic glaciers in Alaska, Greenland and Svalbard (total 15 sites on seven glaciers), OTU1, Oscillatoriales cyanobacterium showing 99% similarity with *Phormidesmis*, was ubiquitous and the most abundant taxon from nearly all investigated sites. In Southern and Northern Asian alpine cryoconites (21 sites on six glaciers), on the other hand, *Microcoleus* OTU4 and Oscillatoriales OTU0 (*Leptolyngbya*, Uetake *et al*., 2019) were found to be prominent cyanobacterial taxa. *Phormidesmis* was occasionally found abundant in these Asian alpine cryoconites, but its detection was intermittent, and the population was also significantly less than that of the dominant Oscillatoriales genera. These results are partly supported by other studies that *Phormidesmis priestleyi* was the primary bacterial species in cryoconites sampled from 38 sites on 10 glaciers in Greenland (Uetake *et al*., 2019) and 37 sites across an ice cap in Central Svalbard (Gokul *et al*., 2016).

**Table 1:** Geographical dominance of Oscillatoriales in cryoconites and OPGs.

Origins of Cryoconites and OPGs	Dominant	Occasionally abundant
Arctic cryoconites	*Phormidesmis* OTU1	OTU0 (*Leptolyngbya*)^a^, OTU7
Asian alpine cryoconites	*Microcoleus* OTU4 OTU0 (*Leptolyngbya*)^a^	OTU8 (Leptolyngbyaceae)^b^ *Phormidesmis* OTU1
Antarctic cryoconites	OTU7 OTU8 (Leptolyngbyaceae)^b^
European HS‐OPGs	*Microcoleus*	*Leptolyngbya*, *Plectonema*
U.S. HS‐OPGs	*Microcoleus*, *Tychonema*	
French SBR‐OPGs	*Microcoleus*	Unclassified Oscillatoriales
U.S. SBR‐OPGs	*Microcoleus*	

The table is generated based on the review of phylogenetic studies conducted on cryoconites and OPGs (Edwards *et al*., 2011; Chrismas *et al*., 2016; Gokul *et al*., 2016, 2019; Milferstedt *et al*., 2017; Segawa *et al*., 2017, 2020; Stauch‐White *et al*., 2017; Uetake *et al*., 2019). Operational taxonomic units (OTU) shown in the table are OTUs in the study of Segawa *et al*. (2017).

HS‐OPGs: hydrostatically formed OPGs; SBR‐OPGs: OPGs produced in sequencing batch reactors.

^a^ Uetake *et al*. (2019).

^b^ Segawa *et al*. (2020).

In OPGs, the genus *Microcoleus* has been found as a prime cyanobacterial taxon regardless of geographic origins (Table 1). A study conducting nine hydrostatic cultivations using activated sludge from four wastewater treatment plants in Europe and the U.S. found that *Microcoleus* was the major cyanobacterial clade, comprising up to 99% of cyanobacteria (Milferstedt *et al*., 2017). This was also the case for OPGs produced in both the French and the U.S. OPG reactors treating local municipal wastewater – these reactors were both seeded with hydrostatically formed OPGs. Nevertheless, the same study also showed that other genera were occasionally dominant in OPGs (Table 1). Some European hydrostatic OPGs showed an unclassified Oscillatoriales genus being more abundant than *Microcoleus*. Other investigation with five hydrostatic cultivations with activated sludge from one U.S. wastewater treatment facility showed that the genus *Tychonema* was most abundant in the formed OPGs (Stauch‐White *et al*., 2017). *Tychonema* and *Microcoleus* belong to the same family, Microcoleaceae.

The enrichment of Oscillatoriales in hydrostatic OPGs is remarkable because their presence in activated sludge inoculums was in very low abundances, frequently undetected (Milferstedt *et al*., 2017). Also in reactors, the enrichment of Oscillatoriales in OPGs growing in size is apparent. As the size of OPGs increases, the density of cyanobacteria, analysed by the quantity of phycobiliproteins and microscopy, increases (Abouhend *et al*., 2020). Similarly, a study analysing bacterial community based on various size groups of Greenlandic cryoconites found that although *Phormidesmis* were already abundant in small‐size (<250 μm) granules they got further enriched as the granule size increased (Uetake *et al*., 2019). Common in cryoconites and OPGs, the granule size increase occurs with the appearance of concentric cyanobacterial layers. Studies on OPGs and cryoconites found consistent results that outer layers of filamentous cyanobacteria were mainly present in granules greater than certain sizes (Abouhend *et al*., 2020; Segawa *et al*., 2020). In smaller granules, Oscillatoriales were found throughout the granules, indicating photic limited distribution of these cyanobacteria.

Two bacterial phyla, Proteobacteria and Bacteroidetes, are also abundant and ubiquitous in cryoconites and OPGs (Edwards *et al*., 2014; Segawa *et al*., 2014, 2020; Musilova *et al*., 2015; Stibal *et al*., 2015; Cameron *et al*., 2016; Milferstedt *et al*., 2017), suggesting their role in sustaining these microbial ecosystems. However, unlike Cyanobacteria, their taxonomic assignments at class and suborder levels in both granule types are highly variable. Similarly, the dominant cyanobacteria *P*. *priestleyi* in Arctic cryoconites were not correlated with other bacterial taxa (Gokul *et al*., 2016; Uetake *et al*., 2019). Furthermore, based on the RNA‐based 16S rRNA analyses, Cyanobacteria were more prominent than these two and other bacterial phyla in Asian alpine cryoconites (Segawa *et al*., 2014, 2020) and frequently in Arctic cryoconites (Stibal *et al*., 2015; Cameron *et al*., 2016). Excluding cyanobacteria, no core bacterial community was found from hydrostatic OPGs from European and the U.S. cultivations (Milferstedt *et al*., 2017). Consequently, bacterial community other than Oscillatoriales are considered general habitants in OPGs and cryoconites, reflecting local and regional effects (Edwards *et al*., 2014; Cameron *et al*., 2016; Milferstedt *et al*., 2017; Uetake *et al*., 2019). Nevertheless, a study contended that Actinobacteria should be the microbial keystone taxa for cryoconites due to their high connectivity in modular community structure found in cryoconites from an ice cap on Svalbard (Gokul *et al*., 2016). This actinobacterial group was also thought to be responsible for humification of organic matter, posing dark colour in cryoconites.

Gliding motility is an important trait of Oscillatoriales – heterocystous cyanobacteria are usually immotile (Stal, 2007) – and therefore it may be a phenotypic characteristic required for granulation of cryoconites and OPGs (Milferstedt *et al*., 2017), henceforth photogranulation. Motility allows Oscillatoriales to move toward or away from the light source, which is an essential substrate but also a stressor (Castenholz, 1968; Stal, 2012). Oscillatoriales glide over a solid surface, including other trichomes (Castenholz, 1968; Hoiczyk and Baumeister, 1995; Stal, 2012). For this to occur, Oscillatoriales are known to secrete significant amounts of extracellular slime or mucilage (Hoiczyk and Baumeister, 1995). Copious amounts of mucilage, often in tubes, around or left behind filamentous cyanobacteria have been reported for both cryoconites and OPGs (Takeuchi *et al*., 2001; Kuo‐Dahab *et al*., 2018). Hence, the movement of Oscillatoriales with their exuded EPS is thought to enhance binding of minerals and also attract bacteria that use cyanobacterial metabolites, including EPS and oxygen, promoting agglomeration in both cryoconites and OPGs (Takeuchi *et al*., 2001; Hodson *et al*., 2010; Langford *et al*., 2010; Kuo‐Dahab *et al*., 2018; Trebuch *et al*., 2020).

**Fig. 4:**
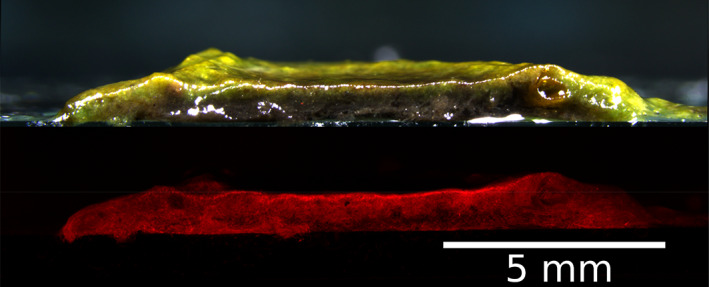
Microbial mat resulting from hydrostatic cultivation with an activated sludge inoculum. This cultivation resulted in a flat mat instead of a spherical OPG. Top: Cross‐section of the microbial mat by light microscopy. Bottom: autofluorescence of cyanobacterial phycocyanin in the same cross‐section as in the top. Photo Credit: Kim Milferstedt and Jérôme Hamelin, INRAE, Laboratoire de Biotechnologie de l'Environnement (LBE).

Nevertheless, the enrichment of Oscillatoriales and their motility is insufficient to explain how they form spherical granules. In natural environments, from rivers and lakes in temperate regions to those in the Arctic and Antarctica, Oscillatoriales are often dominant in solid‐anchored microbial mats (Tang *et al*., 1997; Seckbach and Oren, 2007; Quesada and Vincent, 2012; Stal, 2012; Zhang *et al*., 2015; Tee *et al*., 2020). Castenholz also reported that in hot‐spring microbial mats dominant with *Oscillatoria terebriformis*, interwoven trichomes were free moving (Castenholz, 1968). Concurrently, hydrostatic cultivation of the same activated sludge inoculum occasionally produces flat mats rather than spherical OPGs (Downes, 2019; Joosten *et al*., 2020), although mats were enriched with Oscillatoriales (Fig. 4). In a related context, cryoconites in Antarctic cryoconite holes tend to be poorly granulated compared to alpine and Arctic counterparts (Smith *et al*., 2016). The coexistence of ‘filamentous OPG’ along with spherical OPG in reactors has also been observed (Ouazaite *et al*., 2021). A cryoconite study documented that mashed cryoconites did not return granular despite the enrichment of filamentous cyanobacteria (Takeuchi *et al*., 2001). These examples of the literature suggest other causality in addition to or other than Oscillatoriales that is required for photogranulation.

## Selection pressures for photogranulation

Microbial granulation occurs in widely varying environments and under diverse microbial metabolisms. In environmental engineering systems, granular processes have been used for methanogenic wastewater treatment (Lettinga *et al*., 1980), aerobic wastewater treatment (Mishima and Nakamura, 1991; Morgenroth *et al*., 1997), removal of nitrogen via anaerobic ammonia oxidation (Dapena‐Mora *et al*., 2004), etc. Nevertheless, microbial granulation is rare – otherwise, granules would be as ubiquitous as biofilms attached to substrata – suggesting that only selected conditions allow the phenomenon to occur. Growing granular, OPGs and cryoconites may share selection pressures with other granules. Furthermore, high similarity between OPGs and cryoconites renders an expectation that types of selection pressures and stresses inducing such selective forces would be the same or highly related.

Conventional wisdom is that hydrodynamic shear (Liu and Tay, 2002, 2004; Chen *et al*., 2007; Wu *et al*., 2020) and hydraulic selective pressure (HSP) (i.e. selective washout) (Lettinga *et al*., 1980; Mishima and Nakamura, 1991; Morgenroth *et al*., 1997; Beun *et al*., 1999; Qin *et al*., 2004; Liu and Tay, 2015) are vital to form granules. The tangential shear stress exerted on microbial aggregates and enhanced aggregate–aggregate collision are known to be essential for granulation (Liu and Tay, 2002; Chen *et al*., 2007). Not only for granules used in built systems but for cryoconites (Takeuchi *et al*., 2001; Langford *et al*., 2010) and other natural granules, such as lake algal balls (also known as Marimo) (Boedeker and Immers, 2009), shear has been viewed as a critical component for their physical shaping. HSP – while shear can be still present in non‐granulating systems – provides strong impetus for microbial cells to granulate, thereby avoiding cell washout. For example, reactors seeded with activated sludge enriched with *Candidatus* Accumulibacter phosphatis (Accumulibacter), a polyphosphate accumulating organism, and operated with little HSP continued to support the floccular growth but those with HSP resulted in granulation (Barr *et al*., 2016). The same study further showed that divergence between the floccular and granular systems occurred with substantial change in microbial physiology, revealed by metaproteomics analysis, despite Accumulibacter's continued dominance in both systems. This HSP has been the basis for primary use of sequencing batch reactor (SBR) for engineered granule processes in which selection pressure can be induced by short settling and discharging unsettled biomass out of the systems (Morgenroth *et al*., 1997; Beun *et al*., 1999).

The formation of OPGs by hydrostatic batch, however, defies these dogmas since the hydrodynamic shear must be negligible and there is no real HSP established (Fig. 3A and B). Nevertheless, like other engineered granules, OPGs are also produced in SBRs (Fig. 1C) under the incidence of hydrodynamic shear as well as HSP. Typical mixing conditions in SBRs for OPGs induce shear, computed as velocity gradient, at 40–60 s^−1^ (Milferstedt *et al*., 2017; Abouhend *et al*., 2018). Hydrodynamic shear also showed strong influence on OPGs' size development in SBRs (Abouhend *et al*., in preparation). The reactor with the lowest shear studied, 15 s^−1^, allowed OPG to increase up to 5.5 mm in diameter. On the other hand, the reactor with the highest mixing intensity, 140 s^−1^, did not allow OPG to grow beyond 2.5 mm. For HSP, settling time was 10–15 min (Milferstedt *et al*., 2017; Abouhend *et al*., 2018; Ansari *et al*., 2019), in good agreement with other granular processes (Morgenroth *et al*., 1997; Beun *et al*., 1999; Dapena‐Mora *et al*., 2004; Iorhemen *et al*., 2020) and substantially shorter than settling time (a few hours) employed in the activated sludge process. Hence, granulation of OPGs occurs in a wide spectrum of hydraulic conditions, from the typical spectrum of hydrodynamic shear and HSP for engineered granular processes to where these are virtually nil.

**Fig. 5:**
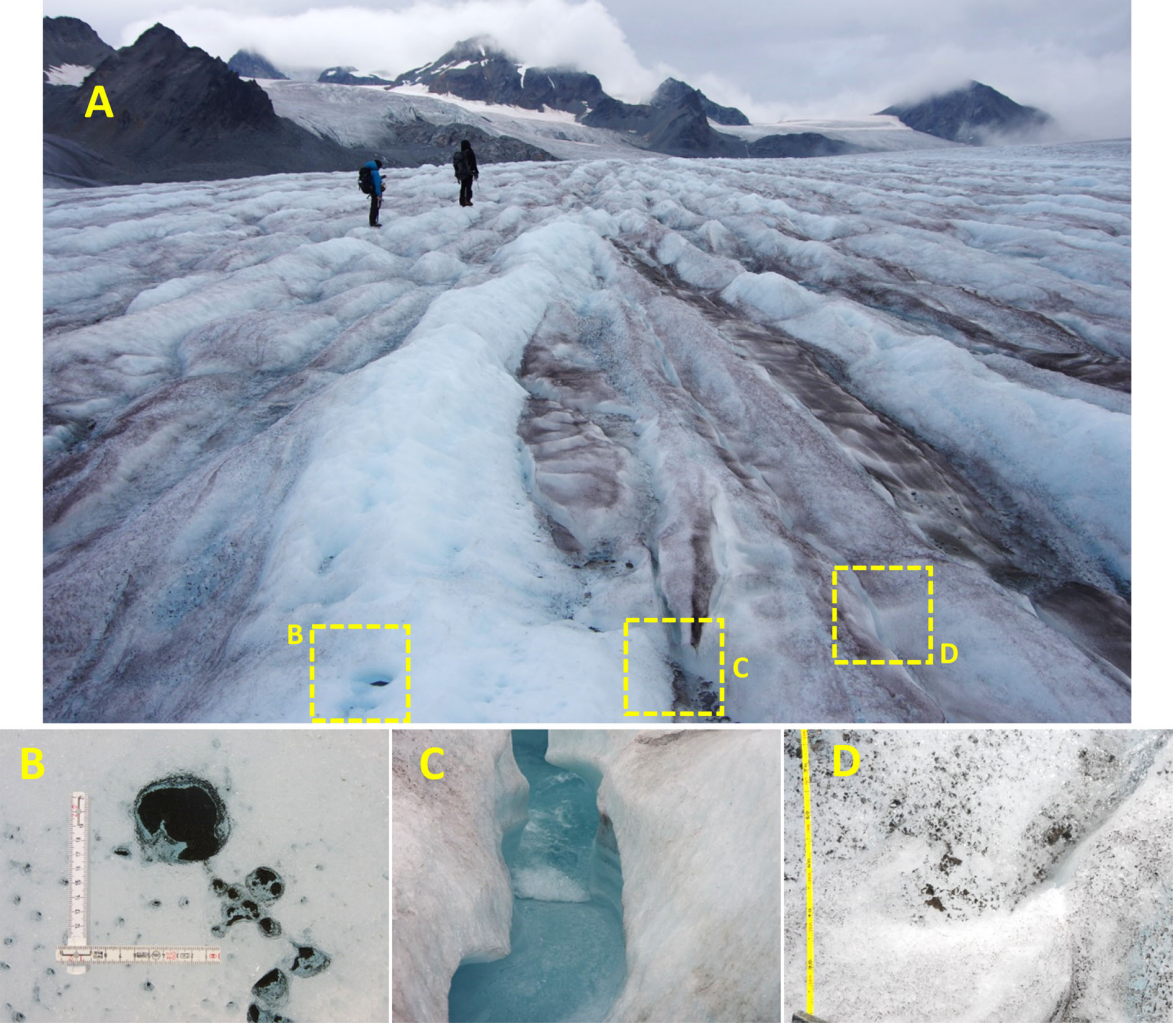
Glacier surface with different hydraulic conditions. A. Ablating glacier ice surface (Gulkana Glacier, Alaska, August 2019). B. Cryoconite holes. C. Meltwater stream. D. Ice surface with distributed cryoconites.

**Fig. 6:**
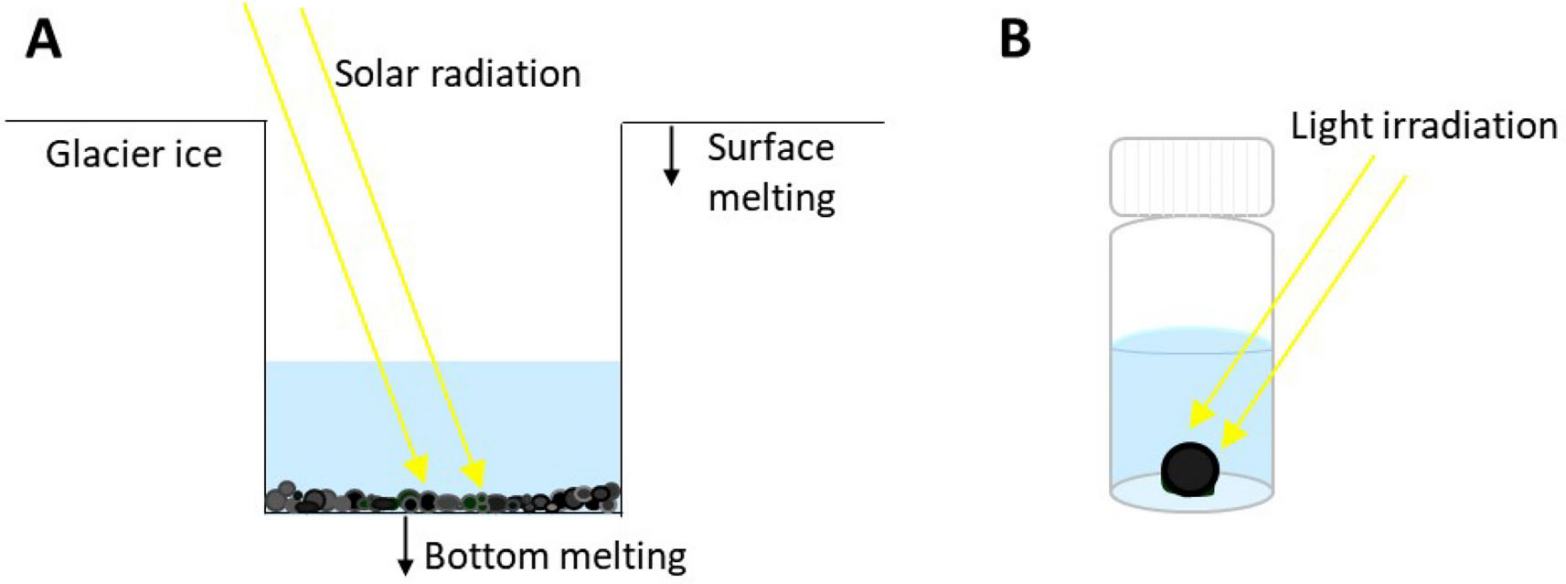
Schematic of a single cryoconite hole and a hydrostatic OPG system. A. A melt hole on an alpine and Arctic glacier surface containing cryoconites at the bottom beneath quiescent water. Cryoconite holes in Antarctica are typically entombed with ice lids. Cryoconite holes are usually interconnected, and water may seep into and out of the holes via porous ice structures. Nevertheless, holes are thought to provide quiescent water conditions. B. A hydrostatic batch vial where a sphere‐like OPG is developed from an activated sludge inoculum.

Characterizing hydraulic conditions of glacier surfaces and understanding its effect on granulation of cryoconites would obviously be more complicated than those for OPGs. Glacier surfaces, particularly ablating Arctic and mountain ice surfaces where cryoconites grow, show three prominent physical features: ice surface, cryoconite holes and meltwater streams (Fig. 5). Each of them has distinct hydraulic conditions, which are most dynamic for meltwater stream, intermediate for ice surface and least for cryoconite holes (Edwards *et al*., 2011). Literature shows that cryoconite holes in alpine glaciers, in which ice surface is typically steeper than that in Polar glaciers, seldom persist through an entire ablating season (Hodson *et al*., 2008; Franzetti *et al*., 2017). Cryoconites in collapsing holes are subjected to dispersal to the ice surface and may undergo subsequent rounds of hole formation and hole destruction or washout via meltwater, which should all involve certain levels of hydrodynamic shear and HSP. Nevertheless, there would be always some periods for cryoconites to sit at the bottom of the hole under quiescent water, which may be analogous to the OPG's hydrostatic batch (Fig. 6). Continual flow of water into and out of cryoconite holes via porous ice structures and, thus, the interconnectivity and dynamic hydraulic conditions of cryoconite holes is now known (Cook *et al*., 2016b). Nevertheless, frequent reports showing that water in cryoconite holes appear nearly static (Yoshimura *et al*., 1997; Takeuchi *et al*., 2001) make us infer that the influence of shear on cryoconites inside the holes is insignificant. Extended period with little or minimal shear can be expected for cryoconites residing in melt holes in flat ice surface, which is more common for Polar ice sheets. Indeed, abundant cryoconites tend to be located dispersed on ice surfaces in mountain glaciers while they are more limited inside the holes for Polar ice sheets and glaciers (Takeuchi, 2002; Takeuchi *et al*., 2018).

Thus, some commonality between OPG and cryoconite may be seen from the hydraulic condition perspective, and this can be further extended with its effect on their physical properties. Hydrostatically formed OPGs are more porous and permeable than OPGs generated in SBRs (Gikonyo, 2020). When the outer layer is removed from hydrostatically formed OPG, the internal biomass often drips out (Milferstedt *et al*., 2017). On the other hand, OPGs produced in reactors are more tightly held together and more spherical, which likely results from their growth in the presence of shear and HSP. For cryoconites, there has been some consensus among researchers that alpine cryoconites are more spherical and tightly aggregated than Arctic and Antarctic cryoconites. Hence, cryoconites experiencing more dynamic hydraulic events in alpine glacier surfaces, analogous to OPGs in SBRs, might provide explanations to this observation.

The hydraulic commonalities, however, do not provide clear explanation to the photogranulation mechanisms since they can be countered by a recurring notion: *how can OPGs*, *and possibly cryoconites*, *granulate both with and without hydrodynamic shear or hydraulic selective pressure?* Although cryoconites and OPGs are always under the influence of certain hydraulic conditions, including a hydrostatic realm, this question requires us to look at other environmental stimuli that may impact their formation.

Microbes in perennial cryoconites (Hodson *et al*., 2010; Takeuchi *et al*., 2010; Cook *et al*., 2016a) live experiencing frequent freeze–thaws and long‐term freeze over the winter. It is well accepted that Oscillatoriales in cryoconites and other habitats in the cryosphere are not true psychrophilic but psychrotrophic microbes (Tang *et al*., 1997; Chrismas *et al*., 2016) and must tolerate various cold stresses, including osmotic stress (Poniecka *et al*., 2020). Numerous freeze–thaw cycles can also naturally induce feast‐famine conditions by limiting substrate availability to microbes beyond cold stresses. These cryoconite microbes also must be protected from high‐intensity sunlight, including UV, radiating onto glacier surfaces (Bagshaw *et al*., 2016). The limited resource environment, including carbon, nitrogen, phosphorous, sulfur and iron, can also induce microbial stress. For example, the level of dissolved nitrogen in cryoconite holes was an order of magnitude lower than that in precipitation or surface meltwater (Segawa *et al*., 2014). Furthermore, the mass of *Phormidesmis* in Arctic cryoconites was correlated with the amount of minerals, implying nutrient limitations (Uetake *et al*., 2016).

A preliminary study in the author's laboratory showed that OPGs maintained granular and treatment functions (oxygen production and COD removal) even after numerous freeze–thaw cycles, indicating that Oscillatoriales in OPGs can also persist in cold stresses like those in cryoconites. However, OPGs have been formed at typical room temperatures and no reports so far have indicated that cold stresses are required for granulation of OPGs. Then, a question may arise: *Is cold stress not essential for photogranulation?* In hydrostatic cultivation of OPGs, depletion of dissolved inorganic nitrogen (DIN) (Stauch‐White *et al*., 2017; Kuo‐Dahab *et al*., 2018; Ansari *et al*., 2021) and labile iron (Ansari *et al*., 2021) – the latter includes dissolved and easily extractable iron – has been documented, suggesting microbial stresses related to limitation of these essential resources. In reactor operation of OPGs, however, a significant level of DIN remains in the effluent (Abouhend *et al*., 2018; Ansari *et al*., 2019; Trebuch *et al*., 2020) – the fate of iron in reactor operation has not yet been reported – thus, OPG granulation still occurred even when nitrogen was sufficiently available. *Is nitrogen limitation not a core requirement for photogranulation?* In terms of phosphorous, some sets of hydrostatic cultivation of OPGs showed depletion while other sets, as well as reactor operation, showed a plenty of phosphate remaining in water (Stauch‐White *et al*., 2017;Kuo‐Dahab *et al*., 2018; Ansari *et al*., 2021). In fill‐and‐draw operation in SBR for OPGs, repetitive feast‐famine conditions occur, and this repetitive feast‐famine cycle has been suggested as a core selection pressure for microbial granulation (Liu and Tay, 2008; López‐Palau *et al*., 2012; Corsino *et al*., 2017; Sun *et al*., 2021). In hydrostatic cultivation of OPGs, which is single batch, feast‐famine cycles are very unlikely to happen.

So, what seems to emerge is not a universal stressor or stressors governing granulation but various stressors under which each cryoconite and OPG system manifests. In a hydrostatic cultivation of OPGs, OPG granulates without experiencing hydrodynamic shear, HSP and feast‐famine cycles but with other stresses, including DIN and Fe limitation and light stress. OPGs in reactors treating wastewater, on the other hand, encounter no limitation of nitrogen but shear stress, HSP and repetitive feast‐famine pressures. Cryoconites on Arctic ice sheets and Asian alpine glaciers would experience different degrees of physical and chemical stresses, including solar radiation and hydraulic events (Segawa *et al*., 2017). And of course, cold stresses are something that microbes in cryoconites must deal with but not for those in OPGs. Despite all these (and more) variations, we see that granular products form in high similarities with the enrichment of the order Oscillatoriales in Cyanobacteria.

## Arrays of stresses and their goldilocks interactions for photogranulation

Hydrostatic OPGs, SBR OPGs, cryoconites in melt holes, cryoconites on bare ice surfaces, cryoconites in alpine glaciers as well as in Polar glaciers all are under certain stress fields but there is no universal ‘external’ cause for their granulation. The more we look the more divergence we find. This probably makes sense because environmental factors associated with the niches of cryoconites and OPGs, and their intravariations, are already vastly different. *So*, *how can photogranulation occur under these barely related environmental conditions?*

Recently, another new way to produce OPGs has been reported. The report shows the hydrodynamic (not hydrostatic) batch of OPGs, generating OPGs directly from activated sludge inoculums with application of mixing (Gikonyo *et al*., 2021). This study conducted 27 batches that varied in the level of three forms of energy, shear stress, light energy and chemical energy, to activated sludge. It found that granulation with enrichment of Oscillatoriales only occurred under certain combinations of these three stresses. For example, granulation was observed in cultivations with ×4 dilute activated sludge inoculum combined with ‘low light’ and ‘high shear’. However, increase in light intensity in the otherwise same condition led to no observable OPG granulation. Although it was for aerobic granule sludge, researchers predicted with biofilm modelling that granulation would occur under combined effects of shear and substrate levels, and theoretically it is possible to generate aerobic granule sludge also under a non‐mixing condition (Wu *et al*., 2020).

These notions align with recent development in cryoconite and cryoconite hole research. Biocryomorphology, study of ice and microbe interactions and their amplified effects on supraglacial melt, has its roots in understanding interactions among ice surface topography, hydrological processes, melt, microbial processes and the geometry of cryoconite holes (Cook *et al*., 2015, 2016c, 2018). Cryoconite holes as biocryomorphic features are dynamic entities that change their morphologies in response to environmental conditions including light onto and within the ice surface and microbial processes entwined via feedback loops. Hence, it seems quite plausible that the interactive effects of the abiotic and biotic factors – that govern the morphology of cryoconite holes – would govern the morphology of cryoconites themselves, accounting for variations in physical and other properties among cryoconites, especially for those from different geographical sources (e.g. Asian alpine, Arctic, and Antarctic cryoconites).

Altogether, we contend that the zone of photogranulation exists for various combinations of ensembles of environmental conditions in which the magnitude of each condition or stress type also matters. This goldilocks photogranulation notion (Gikonyo *et al*., 2021), therefore, provides a means to explain why highly related photogranular products occur across so diverse yet limited environments.

## Concluding remarks and finding the rules of photogranulation

For cryoconite and OPG granulation to occur, it is evident that Oscillatoriales are selected regardless of environmental conditions. OPGs in hydrostatic batch, hydrodynamic batch, or in SBR operations with entries of wastewater (with its microbiome), all result in selection of this microbial group. Hydrostatic batch of OPGs provides panoramic view on it. The enrichment and dominance of Oscillatoriales occur with turnover of original microbial community (Milferstedt *et al*., 2017) including bloom of microalgae which also occurs before the advent of Oscillatoriales (Kuo‐Dahab *et al*., 2018; Ansari *et al*., 2021). We also know that photogranulation does not require a specific lineage of Oscillatoriales, which means that any members of the subsection III cyanobacteria should be able to form cryoconites and OPGs. Nevertheless, the geographical dominance for cryoconites and the frequent dominance of Microcoleaceae in OPGs suggest that Oscillatoriales adapted to or preselected in regional glaciers (Segawa *et al*., 2017) and wastewater environments get selected and drive the granulation process.

**Fig. 7:**
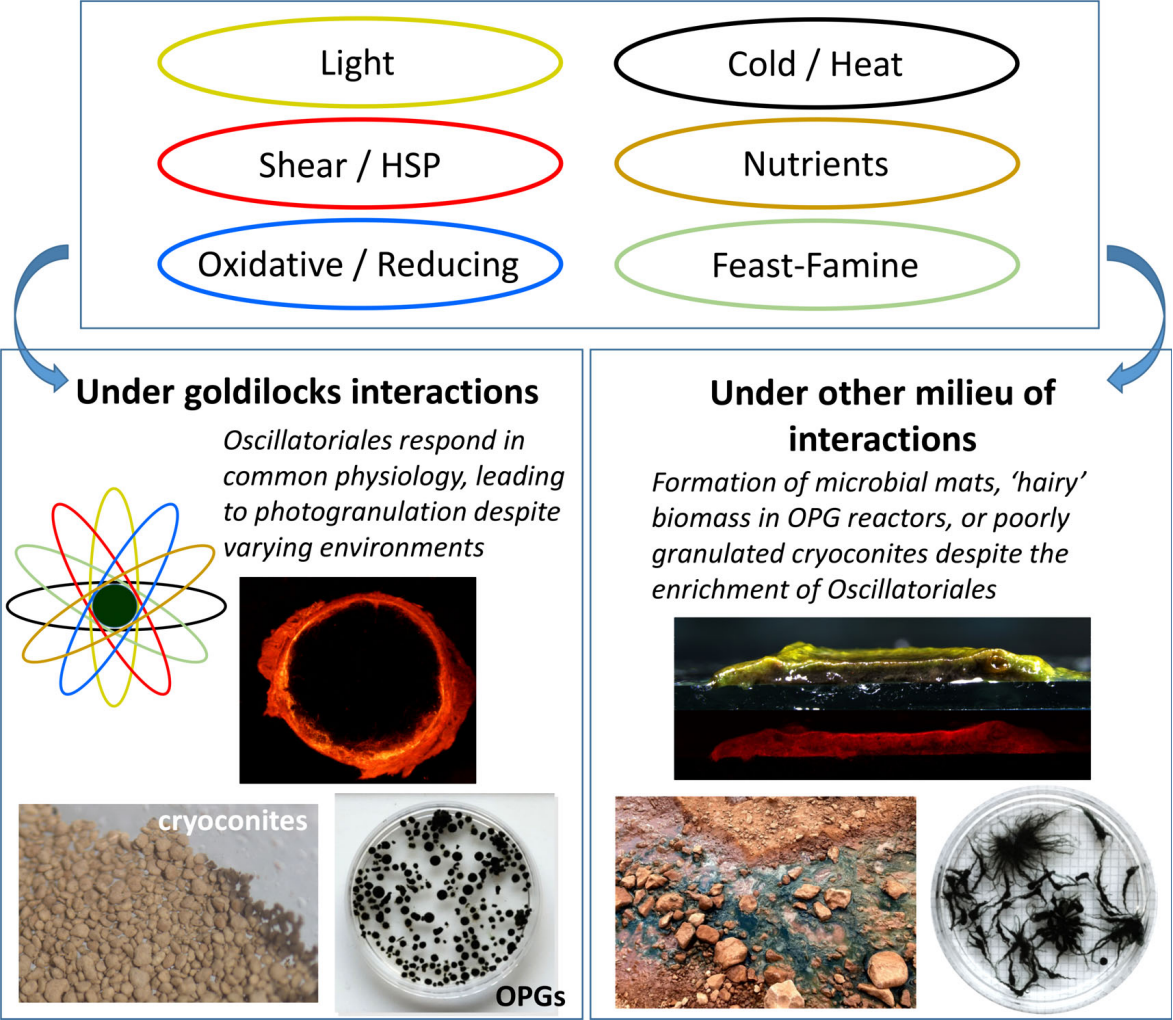
Photogranulation occurring under goldilocks interactions of stresses. Enrichment of Oscillatoriales often occurs in both granular and non‐granular morphologies. The review suggests that Oscillatoriales are granulated by universal physiological processes when they interface with stresses in the goldilocks interactions. The photogranulation interaction zone may be selective or limited – otherwise, cryoconite or OPG‐like granules should be commonplace – yet highly variable accounting for their occurrences under barely related environmental conditions.

The selection of Oscillatoriales itself, however, is not sufficient for photogranulation. In natural environments, microbial mats dominant with Oscillatoriales are ubiquitous. The OPG systems also have shown the formation of mats or streamer‐based filamentous conglomerates. There should be other or additional causation for the phenomenon to occur. For this, we contend that goldilocks interactions of various stresses cause universal physiological responses from Oscillatoriales, which select for their manifestation in spheroidal aggregates (Fig. 7). The blends of external stimuli causing their granulation must be highly variable. Yet, regardless of these variations, Oscillatoriales may respond with common physiological mechanisms, leading to the highly shared observable properties. Consequently, in order to enhance our understanding of the photogranulation phenomenon, efforts should be directed toward identifying common physiology rules, including phenotypic effects, rather than common cohort of external causations – the latter of which unlikely exists for the entire photogranulation systems.

A few metagenomic studies have been done on cryoconites (Edwards *et al*., 2013; Franzetti *et al*., 2016; Hauptmann *et al*., 2017) – There is none yet for OPGs. A study found functional enrichment of genes in stress response, nutrient cycling and motility, suggesting that these microbial functionalities play role in sustaining cryoconites (Edwards *et al*., 2013). Nevertheless, advancing knowledge of cryoconite granulation would require us to know actual expression and regulation of the genes, since the growth of Oscillatoriales, again, could lead to different phenotypes. Sequencing the genome of *P*. *priestleyi* originating from Arctic cryoconites, Chrismas *et al*.  (2016) proposed that regulation of biosynthesis of EPS could be involved for its cold tolerance as well as granulation of cryoconites. We expect that Oscillatoriales' physiology for dwelling in Polar environments and forming cryoconites is not necessarily coupled, since microbial mats found in these harsh environments are also often dominated by the same cyanobacterial clade (Tang *et al*., 1997; Quesada and Vincent, 2012; Zhang *et al*., 2015).

Metatranscriptomic and/or metaproteomics evaluations will be key to finding Oscillatoriales' physiological properties required for the granulation of cryoconites and OPGs. Cryoconites from different glaciers, different melt holes, or even assorted sizes within the same hole could show diverse transcriptomic and proteomic outfits. However, convergences on genes/proteins, especially those related to stress response and cell surface properties, are expected to emerge among cryoconites – worth noting that expression of proteins related to such physiological properties made granular growth different than floccular growth of activated sludge (Barr *et al*., 2016). Similar prediction is also made for OPGs. Finally, finding the omics convergences between OPGs and cryoconites may provide the opportunity to uncover the rules for photogranulation. When these core physiological traits remain on for Oscillatoriales, we hypothesize that granular products related to cryoconite and OPG would occur regardless of geophysical environments. Indeed, Oscillatoriales‐dominant granular products have appeared in coastal zones of Lake Baikal (Volkova *et al*., 2020) and cultivations of Oscillatoriales isolates, from North Sea microbial mats and intertidal mudflats, in seawater environments (Malin and Pearson, 1988; Brehm *et al*., 2003; Brehm, 2004).

The implications of the photogranulation phenomenon are immense. Cryoconites avoid washout and expand on glacier surfaces, causing further decreases in surface albedo and enhanced melt. OPG presents a new path toward sustainable wastewater treatment with self‐aeration and net autotrophy in compact systems. Finding the rules of photogranulation may enhance the engineering of glaciers and wastewater systems to manipulate their ecosystem impacts.

## Author Contributions

C.P. and N.T. conceived this review paper. C.P. took the lead in writing the manuscript. N.T. contributed to the writing and edited the manuscript.
